# Versatile SI-ATRP Growth of Methacrylate Brushes on Superparamagnetic Iron Oxide Nanoparticles Enables Methotrexate-Mediated Antineoplastic Activity in MCF-7 Cells

**DOI:** 10.3390/pharmaceutics18060691

**Published:** 2026-06-01

**Authors:** Razvan Ghiarasim, Alexandru Rotaru, Cristian-Dragos Varganici, Mariana Pinteala, Narcisa-Laura Marangoci, Ion Tiginyanu, Natalia Simionescu

**Affiliations:** 1 Centre of Advanced Research in Bionanoconjugates and Biopolymers, “Petru Poni” Institute of Macromolecular Chemistry, Romanian Academy, Grigore Ghica Voda Alley 41 A, 700487 Iasi, Romania; ghiarasim.razvan@icmpp.ro (R.G.); rotaru.alexandru@icmpp.ro (A.R.); varganici.cristian@icmpp.ro (C.-D.V.); pinteala@icmpp.ro (M.P.); nmarangoci@icmpp.ro (N.-L.M.); tighineanu.ion@icmpp.ro (I.T.); 2 National Centre for Materials Study and Testing, Technical University of Moldova, 2004 Chisinau, Moldova

**Keywords:** magnetic nanoparticles, surface-initiated atom transfer radical polymerization (SI-ATRP), polymer, drug, breast-cancer cells

## Abstract

**Background/Objectives:** Superparamagnetic iron-oxide nanoparticles (SPIONs) bearing poly(methacrylate) brushes were synthesized via surface-initiated atom-transfer radical polymerization (SI-ATRP) as magnetically responsive nanoplatforms. Three brush architectures, poly(2-hydroxyethyl methacrylate) (PHEMA) and poly(poly(ethylene glycol) methacrylate) with six ethylene-oxide units (PPEGMA6) and ten units (PPEGMA10), were grown from a dopamine-anchored initiator and covalently loaded with methotrexate (MTX). **Methods:** Physicochemical characterization confirmed successful polymer grafting, tunable hydrodynamic size (185–1320 nm before MTX conjugation and 427–694 nm after), retained superparamagnetic properties (22–69 emu g^−1^), and high drug payloads, with PPEGMA6 achieving 131 µg mg^−1^. MTX conjugation induced partial compaction of the polymer shell yet maintained ζ-potentials conducive to colloidal stability. **Results:** In vitro assays showed negligible toxicity toward primary human fibroblasts, whereas MTX-decorated formulations induced a pronounced concentration-dependent cytotoxic effect in MCF-7 breast cancer cells, reaching 69% loss of viability—significantly higher than free MTX. Structure–activity analysis attributes the superior performance of PPEGMA6-MTX to its balanced brush density, high payload, and favorable surface charge. **Conclusions:** These findings demonstrate that precise modulation of polymer brush architecture via SI-ATRP yields SPION-based nanocarriers that integrate MRI visibility and the potential for magnetic guidance and targeted chemotherapy. The PPEGMA6-MTX construct is highlighted as a promising platform for future preclinical investigations.

## 1. Introduction

Cancer remains one of the leading causes of mortality worldwide. According to the GLOBOCAN 2020 report, there were ~19.3 million new cases and almost 10 million deaths in 2020, and the global cancer burden is projected to climb to 28.4 million new cases by 2040 [1]. To meet this challenge, nanomedicine has emerged as a powerful model that couples diagnosis and therapy at the nanoscale. Current nanomedicine strategies fall largely into two classes: (i) theranostic platforms that simultaneously enable imaging-guided therapy, and (ii) nanocarriers that deliver drugs with high precision [2]. Among the wide spectrum of nanocarriers, superparamagnetic iron-oxide nanoparticles (SPIONs) stand out owing to their inherent magnetic-resonance-imaging (MRI) contrast, remote magnetic guidance, scalable aqueous synthesis, and proven biocompatibility [3,4,5,6]. Nevertheless, the unmodified iron oxide surface offers limited colloidal stability and drug-loading capacity. A proven solution is to graft a dense polymer shell that not only stabilizes the colloid but also furnishes a high density of functional groups for drug conjugation and bio-interface engineering [7]. Recent studies have reported various polymer-functionalized iron oxide nanoparticles for drug delivery applications, highlighting their ability to improve drug loading, stability, and biological performance. In this context, polymer-coated magnetic nanoparticles have been widely explored for the delivery of anticancer agents, including doxorubicin [8,9], paclitaxel [10,11], methotrexate [12,13], curcumin [14], and 5-fluorouracil [15], further demonstrating their versatility as nanocarriers. Polymer attachment is typically achieved via two principal routes. In the grafting-to approach, pre-made polymers are tethered to the surface through end-group chemistry, whereas the grafting-from (surface-initiated) strategy grows polymer chains directly from initiators immobilized on the nanoparticle’s surface, achieving markedly higher grafting densities [16]. This method leads to a higher density of polymer obtained on a surface than grafting-to, which translates into the possibility of binding a much higher concentration of molecules of interest. Surface-initiated atom-transfer radical polymerization (SI-ATRP) is particularly attractive because it accommodates a broad palette of methacrylate monomers, while the polymerization initiator is bound to a surface [17], offering exquisite control over the degree of polymerization, architectural complexity, and end-group functionality [18,19,20]. The methacrylate-based polymers obtained by SI-ATRP are used due to their biocompatibility, as well as the swelling property that leads to non-specific interactions with cell membranes [21]. Despite these advantages, the influence of SI-ATRP brush length on subsequent drug loading and biological performance has not been systematically elucidated.

Methotrexate (MTX) is one of the most used drugs with a wide spectrum of clinical applications [22], starting from the administration for the first time in the treatment of childhood leukemia in 1948 [23], becoming a standard drug for the treatment of rheumatoid arthritis [24] to the treatment against breast cancer [25]. MTX is an antimetabolite of folic acid with a complex mechanism of action [26], being internalized in cells through folate receptors that are overexpressed on the surface of tumor cells compared to healthy ones [27], especially through the folate receptor beta (FRβ) [28]. Once inside the cell, methotrexate inhibits the action of dihydrofolate reductase, one of the enzymes involved in the synthesis of purine and pyrimidine bases, critical for DNA synthesis, leading to the disruption of cell proliferation [29]. Due to the dual action of methotrexate (as targeted delivery and therapeutic action), over the last decades, it has been included in a wide range of chemical systems for drug delivery, such as magnetic nanoparticles decorated with polymers [30,31,32,33,34], liposomes [35,36], micelles [37,38] and pro-drugs [39,40].

Herein, we present the first comparative investigation of SPIONs coated with three poly(methacrylate) brushes of progressively increasing degree of polymerization, synthesized via SI-ATRP and subsequently functionalized with MTX. We show that altering brush length systematically modulates MTX loading, colloidal stability and surface charge, and we directly relate these physicochemical parameters to biological responses in normal human fibroblasts and in MCF-7 breast cancer cells. This structure–property perspective provides a rationale for engineering magnetic nanocarriers with a tailored polymer shell that maximizes therapeutic payload while preserving biocompatibility and imaging capability.

## 2. Materials and Methods

### 2.1. Materials

Iron(II) chloride tetrahydrate (FeCl_2_ × 4H_2_O; 99%, Acros Organics, Geel, Belgium), iron(III) chloride hexahydrate (FeCl_3_ × 6H_2_O; Reag. Ph. Eur., >99%, Honeywell Fluka, Charlotte, NC, USA), 3-hydroxytyramine hydrochloride (L-dopamine, 99%, Acros Organics, Geel, Belgium), α-bromoisobutyryl bromide (98%, Sigma-Aldrich, St. Louis, MO, USA), di-sodium tetraborate decahydrate (Na_2_B_4_O_7_ × 10H_2_O, Supelco, Bellefonte, PA, USA), sodium carbonate decahydrate (≥99.0%, Sigma-Aldrich, St. Louis, MO, USA), 2-hydroxyethyl methacrylate (contains ≤ 250 ppm monomethyl ether hydroquinone as inhibitor, 97%, HEMA, Sigma-Aldrich, St. Louis, MO, USA), poly(ethylene glycol) methacrylate (average Mn 360, contains 500–800 ppm MEHQ as inhibitor, PEGMA6, Sigma-Aldrich, St. Louis, MO, USA), poly(ethylene glycol) methacrylate (average Mn 500, contains 900 ppm monomethyl ether hydroquinone as inhibitor, PEGMA10, Sigma-Aldrich, St. Louis, MO, USA), N,N,N′,N″,N″-pentamethyldiethylenetriamine (99%, PMDETA, Sigma-Aldrich, St. Louis, MO, USA), copper (I) bromide (≥98.0%, Honeywell Fluka, Charlotte, NC, USA), copper (II) bromide (99%, Sigma-Aldrich, St. Louis, MO, USA), methotrexate (MTX, pharmaceutical secondary standard, Supelco, Bellefonte, PA, USA), 4-(dimethylamino) pyridine (≥99.0%, DMAP, Sigma-Aldrich, St. Louis, MO, USA), N-N′-diisopropylcarbodiimide (DIC, 99%, Sigma-Aldrich, St. Louis, MO, USA), N,N′-dimethylformamide pure (Lach-Ner, Neratovice, Czech Republic), ammonium hydroxide solution (NH_4_OH 25% in water; Honeywell Fluka, Charlotte, NC, USA), hydrochloric acid (37%, Supelco, Bellefonte, PA, USA), methanol (≥99.8%, Sigma-Aldrich, St. Louis, MO, USA), chloroform (99.0–99.4% (GC), Honeywell Fluka, Charlotte, NC, USA), phosphate buffered saline (PBS, 1X, Sigma-Aldrich, St. Louis, MO, USA), and aluminum oxide 90 active basic (alumina, 0.063–0.200 nm, Merck KGaA, Darmstadt, Germany). Except for the three monomers (HEMA, PEGMA6, and PEGMA10), which were added through an alumina column to remove the inhibitor and held under an inert atmosphere at 20 °C prior to polymerization, all components were utilized as received.

### 2.2. Synthesis of Magnetic Nanoparticles (MNP-OH)

The co-precipitation method, as described by Anghelache et al. [4] in their study, was employed to synthesize unmodified magnetic nanoparticles (MNP-OH). Briefly, (1.72 g, 0.00868 mol) FeCl_2_·4H_2_O and FeCl_3_·6H_2_O (4.70 g, 0.01738 mol) were dissolved in 50 mL of deionized water and stirred at 80 °C under N_2_ for 30 min. Then, 5 mL of 25% NH_4_OH was added until pH 10, forming a black suspension. Stirring continued for another 30 min. The nanoparticles were washed with purified water (5×) to neutral pH, freeze-dried, and stored under an inert atmosphere. The obtained Fe_3_O_4_ nanoparticles were isolated as a dried material (1.75 g), with a residual water content of 2.37%, as determined by thermogravimetric analysis.

### 2.3. Grafting of the ATRP-Type Initiator onto the Unmodified Magnetic Nanoparticles’ Surface (MNP-I)

In a 250 mL flask, 0.55 g of MNP-OH was dispersed in 100 mL of deionized water, over which ATRP initiator (1 g, 0.0018 mol) (synthesized as previously described by us [41]) was added. The suspension was subjected to mechanical agitation, protected from light for 24 h, and then the ATRP initiator-decorated nanoparticles (MNP-I) were washed several times with deionized water and stored at 4 °C.

### 2.4. Polymerization of HEMA, PEGMA6 and PEGMA10 Monomers by SI-ATRP (MNP-PHEMA-OH, MNP-PPEGMA6-OH, MNP-PPEGMA10-OH)

The polymerization of the three monomers was carried out following the molar ratios reported in the literature [42]. For the HEMA-based system, a molar ratio of HEMA/PMDETA/CuBr_2_/CuBr = 200/10/1/3.5 was employed. In a 100 mL flask, HEMA (25 mL, 26.825 g, 0.206 mol), PMDETA (2.150 mL, 1.7849 g, 0.0103 mol), CuBr_2_ (0.230 g, 0.001 mol), and 25 mL of deionized water were introduced. The solution was purged with nitrogen for 30 min to remove dissolved oxygen. Subsequently, CuBr (0.516 g, 0.0036 mol) was added under a nitrogen atmosphere, followed by an additional 30 min nitrogen purging. The resulting polymerization mixture was transferred to a previously degassed aqueous dispersion of MNP-I (4 mg/mL, 25 mL), prepared by nitrogen purging. The reaction was conducted under mechanical stirring at 25 °C for 30 min, under light-protected conditions. The obtained nanoparticles (MNP-PHEMA-OH) were purified by repeated centrifugation and washing with deionized water and stored in an aqueous solution. The same polymerization protocol was applied for PEGMA-based monomers with adjusted molar ratios and reaction conditions. For PEGMA6, a molar ratio of PEGMA6/PMDETA/CuBr2/CuBr = 80/10/1/3.4 was used, employing PEGMA6 (25 mL, 27.625 g, 0.076 mol), PMDETA (1.98 mL, 1.6463 g, 0.0095 mol), CuBr_2_ (0.212 g, 0.00095 mol), and CuBr (0.463 g, 0.00323 mol). The polymerization was performed at 60 °C for 30 min. For PEGMA10, a molar ratio of PEGMA10/PMDETA/CuBr_2_/CuBr = 500/12.5/1/5 was used, with PEGMA10 (25 mL, 27.525 g, 0.055 mol), PMDETA (0.286 mL, 0.238 g, 0.0013 mol), CuBr_2_ (0.024 g, 0.0001 mol), and CuBr (0.078 g, 0.0005 mol). The reaction was carried out at 60 °C for 30 min. All resulting nanoparticles (MNP-PHEMA-OH, MNP-PPEGMA6-OH, and MNP-PPEGMA10-OH) were purified by repeated washing with DMF and subsequently stored in deionized water at 4 °C.

### 2.5. Functionalization of the Three Polymeric Brushes Obtained by SI-ATRP with MTX (MNP-PHEMA-MTX, MNP-PPEGMA6-MTX and MNP-PPEGMA10-MTX)

For functionalization of core-shell nanoparticles with MTX, 100 mg of MNP-PHEMA-OH/MNP-PPEGMA6-OH/MNP-PPEGMA10-OH was dispersed in 50 mL DMF each; then, MTX (0.2 g, 0.44 mmol) and DMAP (0.0053 g, 0.044 mmol) were added, and N_2_ gas was purged for 3 h at 25 °C. Next, DIC (67.4 μL, 0.054 g, 0.44 mmol) was added dropwise over an ice bath, and the solution was subjected to mechanical stirring under N_2_ gas for 72 h at 25 °C. The magnetic nanoparticles with polymeric shell functionalized with MTX (MNP-PHEMA-MTX, MNP-PPEGMA6-MTX and MNP-PPEGMA10-MTX) were washed 3 times with DMF to remove the free MTX, and 3 times with deionized water, and stored in deionized water at 4 °C.

### 2.6. Encapsulation Efficiency (EE) and Drug Loading (DL)

MTX encapsulation efficiency (EE) and drug loading (DL) of MTX were calculated using Equations (1) and (2) [43]. The amount of non-conjugated MTX was determined in the supernatant by UV-Vis spectroscopy at 303 nm.

(1) $$DL\;\left(wt\%\right)=\frac{Weight\;of\;MTX\;in\;MNP}{Total\;weight\;of\;MNP}\times 100$$

(2) $$EE\;\left(wt\%\right)=\frac{Weight\;of\;MTX\;in\;MNP}{Weight\;of\;feeding\;MTX\;}\times 100$$

### 2.7. In Vitro pH-Dependent Release Studies

The in vitro release profile of methotrexate (MTX) from MTX-functionalized nanoparticles was evaluated using a dynamic dialysis method [44]. Initially, the amount of MTX associated with each nanoparticle formulation (MNP-PHEMA-MTX, MNP-PPEGMA6-MTX, and MNP-PPEGMA10-MTX) was determined. Subsequently, the nanoparticle samples, containing 300 µg of MTX dispersed in 32 mL of 1X PBS, together with a control sample of free MTX (600 µg in 32 mL of 1 X PBS), were separately introduced into dialysis membranes (MWCO: 3 kDa). Dialysis experiments were performed against 300 mL of 1X PBS at pH 7.4 and 5.8 to simulate physiological and mildly acidic environments, respectively, maintaining a sample-to-medium volume ratio of 32 mL:300 mL (~1:9.4). The release studies were conducted at 37 °C under continuous stirring. At predetermined time intervals (1, 2, 4, 8, 12, 24, 48, and 72 h), aliquots were collected from the external release medium and replaced with an equal volume of fresh buffer to maintain sink conditions. The concentration of released MTX was quantified using UV-Vis spectroscopy at its characteristic absorption maximum (λ_max_ = 303 nm), based on calibration curves constructed separately at pH 7.4 and 5.8 (Figure S2), to account for pH-dependent variations in MTX absorbance. All measurements were performed in triplicate. The cumulative MTX release was expressed as a percentage and calculated according to the following equation [44]:

$$Cumulative\;MTX\;release\;\left(\%\right)=\left(\frac{Wt}{W}\right)\times 100,$$

where W_t_ represents the amount of MTX released at time t, and W is the total amount of MTX initially associated with the nanoparticles.

### 2.8. Characterization

The FTIR spectra were determined using an IRTracer-100 FTIR spectrometer (Shimadzu, Kyoto, Japan) with the attenuated total reflection (ATR) module, 100 scans, resolution 4 cm^−1^ and in the spectral range 4000–400 cm^−1^.

Absorption spectra in the ultraviolet-visible (UV-vis) range of the magnetic nanoparticles were recorded using a Lambda 35 spectrophotometer (Perkin Elmer Inc., Waltham, MA, USA) with a slit of 1.0 in the wavelength range of 200–700 nm. All samples involving magnetic nanoparticles were measured at a concentration of 50 µg nanoparticles/ mL in PBS pH 7.4, and the absorption spectrum of free MTX was determined at a concentration of 10 μg MTX/mL in PBS pH 7.4.

Thermogravimetric analysis curves were recorded using STA 449 F1 JUPITER (Netzsch, Selb, Germany) equipment. Temperature and sensitivity calibrations in the 30–700 °C range were carried out using indium. Measurements were conducted between 30 and 700 °C at a heating rate of 10 °C per minute and a flow rate of 50 mL per minute in a dry nitrogen environment. The data was processed using NETZSCH Proteus^®^ 4.2 software (Netzsch, Selb, Germany).

The saturation magnetizations of the nanoparticles were measured at 25 °C on a Series 8600 VSM (LakeShore Cryotronics, Westerville, OH, USA) with an applied demagnetization of 20 kOe, and magnetic properties were recorded up to a maximum field of 25 kOe (2.5 Tesla).

Hydrodynamic diameters of nanoparticles were determined by Dynamic Light Scattering (DLS) using a Delsa Nano C Submicron Particle Size Analyzer (Beckman Coulter, Brea, CA, USA) at a scattering angle of 165° and a concentration of 50 µg nanoparticles/mL in PBS pH 7.4 in triplicate with 70 iterations each, also calculating standard deviations.

The zeta potential corresponding to samples involving magnetic nanoparticles was measured using a Delsa Nano C Submicron Particle Size Analyzer (Beckman Coulter, Brea, CA, USA) with a Flow Cell Module at a concentration of 50 µg nanoparticles/mL in PBS pH 7.4. The zeta potentials were measured for 5 points each with 10 iterations, and means and standard deviations were calculated.

The morphology of the samples was studied using a Verios G4 UC Scanning Electron Microscope (Thermo Fisher Scientific, Waltham, MA, USA) in STEM mode at 30 kV with a STEM 3+ detector (Bright-Field Mode). The samples (MNP-OH, MNP-I, MNP-PHEMA-OH, MNP-PPEGMA6-OH, MNP-PPEGMA10-OH, MNP-PHEMA-MTX, MNP-PPEGMA6-MTX and MNP-PPEGMA10-MTX) dispersed in deionized water (12.5 µg nanoparticles/mL) were deposited on carbon-coated copper grids with a mesh size of 300, and the solvent was removed under vacuum.

### 2.9. In Vitro Cytotoxicity

Human gingival fibroblasts (HGFs) and breast adenocarcinoma cells (MCF-7, both from CLS Cell Lines Service GmbH, Eppelheim, Germany) were grown in DMEM medium with low glucose, GlutaMAX™ Supplement and pyruvate, 5% fetal bovine serum (FBS, both from Gibco, Thermo Fisher Scientific, Waltham, MA, USA) and 1% Penicillin-Streptomycin-Amphotericin B (10 K/10 K/25 μg, Lonza, Basel, Switzerland).

Cytotoxicity of nanoparticles was assessed using the CellTiter-Glo^®^ Luminescent Cell Viability Assay (Promega, Madison, WI, USA), according to the manufacturer’s instructions. Cells were seeded into 96-well tissue culture-treated opaque white plates (50,000 cells/mL—HGF, 70,000 cells/mL—MCF-7) and allowed to adhere overnight. Next, cells were incubated with fresh complete medium (control), nanoparticles or MTX solutions for 72 h. Polymer-coated MNPs lacking MTX (MNP-PHEMA-OH, MNP-PPEGMA6-OH, MNP-PPEGMA10-OH) were tested at concentrations equivalent to those used for MTX-functionalized polymer-coated MNPs (0–2.5 µg MTX/mL). The luminescence readings were conducted on a FLUOstar^®^ Omega microplate reader (BMG LABTECH, Ortenberg, Germany). The experiments were carried out in triplicate, and the viability of treated cells was expressed as a percentage of the viability of control cells. Data were represented as means ± standard error of the mean.

## 3. Results and Discussion

**Scheme 1:**
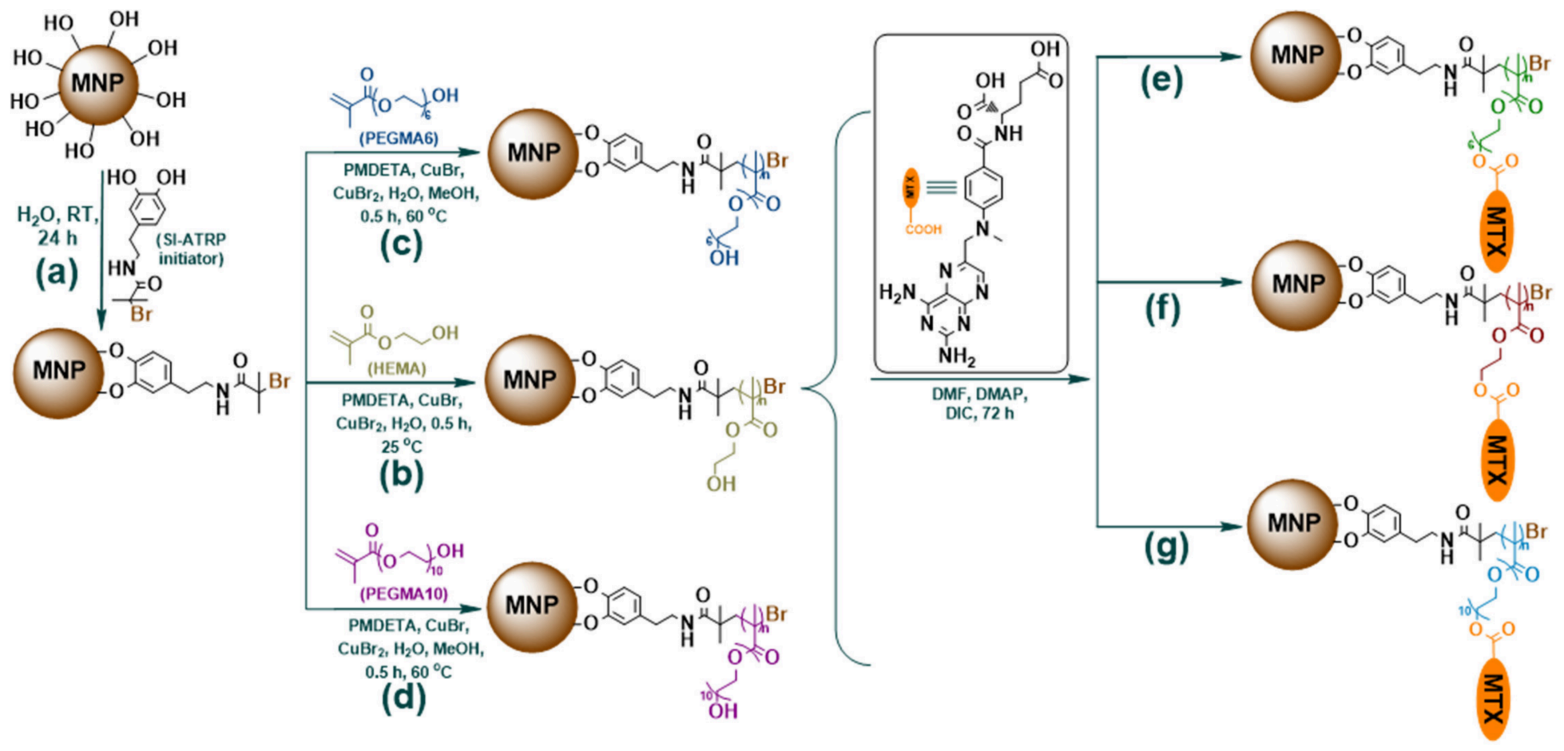
Synthesis steps for the preparation of three methotrexate-based nanocarriers: (**a**) covalent binding of the catechol residue from the structure of the ATRP-type initiator to the surface of the magnetic nanoparticles; polymerization of the monomer HEMA (**b**), PEGMA6 (**c**) and PEGMA10 (**d**) by SI-ATRP; (**e**) functionalization with MTX of PPEGMA6 (**e**), PHEMA (**f**) and PPEGMA10 (**g**) polymers. RT—room temperature; PMDETA-N,N,N′,N″,N″—pentamethyldiethylenetriamine; MeOH—methanol; DMF—dimethylformamide; DMAP—4-dimethylaminopyridine; DIC—N,N′-diisopropylcarbodiimide; MTX—methotrexate.

Magnetic iron oxide nanoparticles (MNPs) were synthesized via the aqueous co-precipitation of FeCl_2_ and FeCl_3_, yielding quasi-spherical cores with an average diameter of ~10 nm. Consistent with this method, the pristine particles exhibited a negative ζ-potential value due to surface-exposed hydroxyl groups. To further modify the nanoparticle’s surfaces, a catecholic residue derived from the ATRP polymerization initiator, which is based on L-dopamine, was covalently linked (Scheme 1). The catechol group of the residue formed strong covalent bonds with the hydroxyl groups on the surface of the nanoparticles. Subsequently, three methacrylate-based monomers, with different molar masses and with free hydroxyl groups on the repeating polymeric structural units, were polymerized by SI-ATRP. The presence of hydroxyl groups provided anchor points for conjugating methotrexate (MTX) via carbodiimide-mediated esterification of the drug’s γ-carboxylate, yielding a pH-labile ester bond. The conjugation strategy, thus, couples a high drug payload to the possibility of acidic-triggered release in the endo/lysosomal milieu.

Each step in the synthetic sequence (Scheme 1) was verified by complementary physicochemical techniques, and MTX loading was quantified by UV–Vis spectrophotometry using an external calibration curve.

**Figure 1:**
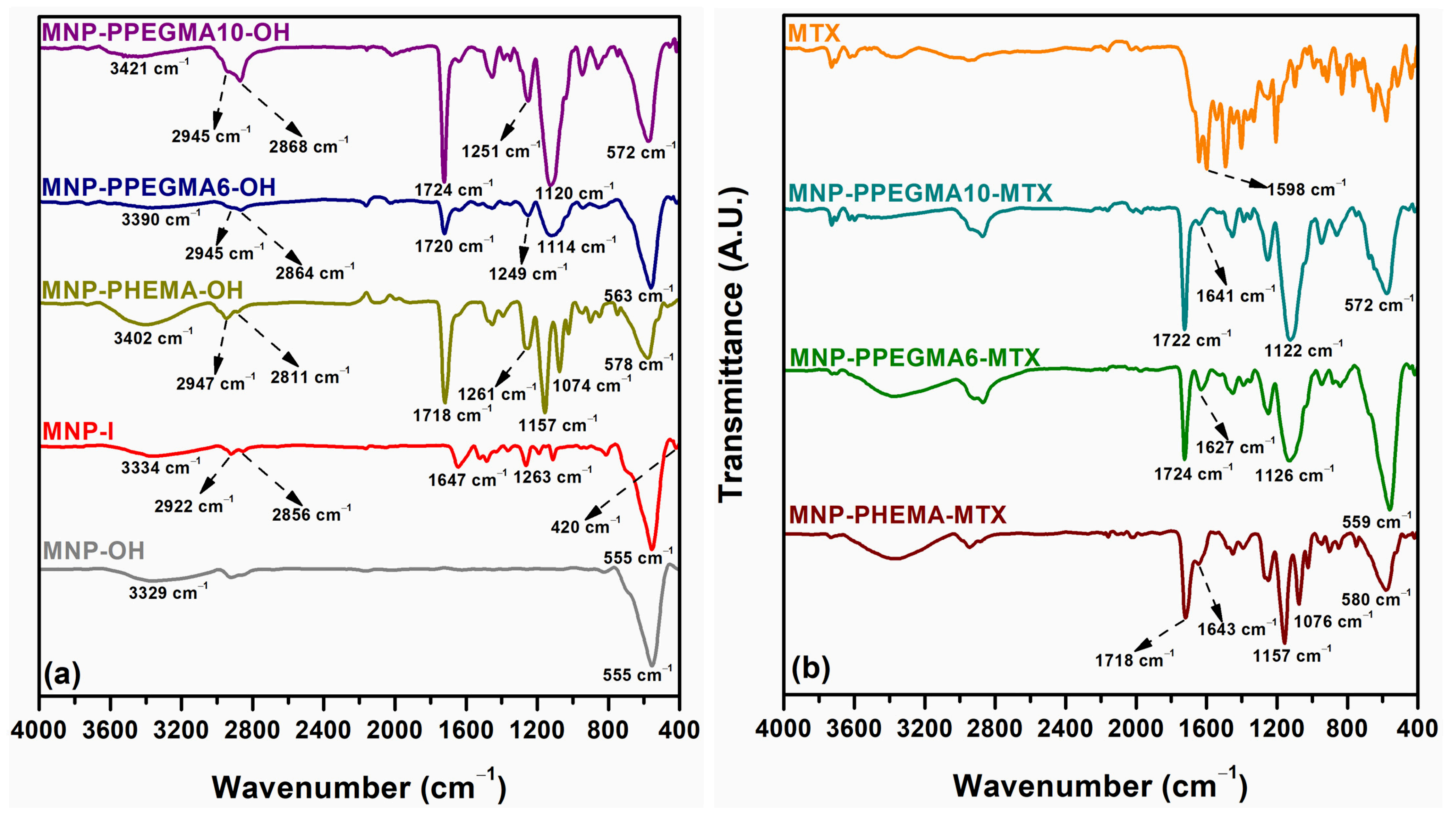
FTIR spectra of: (**a**) unmodified magnetic nanoparticles (MNP-OH), magnetic nanoparticles functionalized with the ATRP-type initiator (MNP-I), and the three polymers obtained by SI-ATRP (MNP-PHEMA-OH, MNP-PPEGMA6-OH and MNP-PPEGMA10-OH); (**b**) free MTX and the corresponding MTX-conjugated formulations (MNP-PHEMA-MTX, MNP-PPEGMA6-MTX and MNP-PPEGMA10-MTX). A.U.—arbitrary units.

Surface modifications of the nanoparticles were monitored by FTIR spectroscopy for both the unmodified MNPs and the polymer-coated MNPs (Figure 1a), MTX-conjugated polymers (Figure 1b), free MTX (Figure 1b), and the three methacrylate-based monomers (Figure S1).

The unmodified magnetic nanoparticles (Figure 1a, MNP-OH) presented (in the FTIR spectrum) two main absorption bands, including the signal at 555 cm^−1^, assigned to the Fe-O stretching vibration in the nanoparticle structure, and the vibration band at a wavenumber of 3329 cm^−1^, corresponding to the broad O-H stretching vibration on the surface of the MNPs [45]. The grafting of the ATRP-type initiator on the surface of the MNPs resulted in the appearance of additional absorption bands characteristic of the initiator (MNP-I).

Thus, in Figure 1a (MNP-I), a characteristic absorption band appeared at 1647 cm^−1^, assigned to the C=C stretching vibration in the structure of the benzene nucleus in the catecholic group, as well as a vibration band at 1263 cm^−1^, representative of the C-C-C asymmetric stretching vibration on the initiator structure. The C-Br stretching signal was highlighted at 420 cm^−1^, the characteristic C-H stretching absorption band at 2856 cm^−1^ (C-H coming from -CH_2_- groups), the characteristic C-H stretching signal at 2922 cm^−1^ (C-H coming from -CH_3_ groups) and, last but not least, the presence of Fe-O from the structure of MNPs at 555 cm^−1^ [41]. Successful HEMA polymerization (Figure 1a, MNP-PHEMA-OH) was confirmed by the disappearance of the C=C stretching vibration band at 1635 cm^−1^ from the monomer structure (Figure S1, HEMA), confirming successful polymerization. The FTIR spectrum of the MNP-PHEMA-OH sample indicated a broad O-H stretching peak at 3402 cm^−1^ from the polymeric repeating units; two vibration bands at 2947 cm^−1^ and 2811 cm^−1^ are characteristic of the C-H asymmetric stretching vibration (-CH_3_ group) and C-H symmetric stretching vibration (-CH_2_- group), an intense band at 1718 cm^−1^ is assigned to C=O stretching vibrations, and three absorption bands at 1261 cm^−1^, 1157 cm^−1^ and 1074 cm^−1^ are assigned to C-C-C stretching vibration, C-O stretching vibration and C-O-C stretching vibration, respectively. The presence of the Fe-O band (578 cm^−1^) further confirms that the band also appears, which indicates that polymer chains were grown on the surface of the MNPs. Polymerization of PEGMA6 and PEGMA10 by SI-ATRP was similarly confirmed by the disappearance of the C=C vibration bands (MNP-PPEGMA6-OH and MNP-PPEGMA10-OH) from 1635 cm^−1^ in the case of PEGMA6 (Figure S1, PEGMA6) and at 1635 cm^−1^ for PEGMA10 (Figure S1, PEGMA10). For the samples MNP-PPEGMA6-OH and MNP-PPEGMA10-OH (Figure 1a), similar absorption bands to those observed for MNP-PHEMA-OH were identified (monomers with the same types of bonds, with the difference coming from the length of the monomeric chain: HEMA has one ethylene glycol unit, PEGMA6 has 6 ethylene glycol units, and PEGMA10 has 10 ethylene glycol units in its structure). The major difference between the samples is the appearance of sharp and well-defined bands in the case of the MNP-PHEMA-OH sample (C-O stretching vibration (1157 cm^−1^) and C-O-C band (1074 cm^−1^), respectively), and in the case of the other two samples MNP-PPEGMA6-OH and MNP-PPEGMA10-OH, respectively; these two bonds are evident in the FTIR spectra as a single broad band (1114 cm^−1^ for MNP-PPEGMA6-OH and 1120 cm^−1^ in the case of MNP-PPEGMA10-OH). This effect is due to the ability of the monomeric units to be flexible (leads to the appearance of broad bands) because of the length of the segments, and for this reason, these bands do not present sharp vibration bands [46].

The functionalization of the polymers grown on the surface of the MNPs with MTX led to the appearance of the specific C=C stretching vibration band from the MTX structure (1598 cm^−1^, MTX, Figure 1b) in the FTIR spectra of MNP-PHEMA-MTX (C=C stretching vibration at 1643 cm^−1^), MNP-PPEGMA6-OH (C=C peak at 1627 cm^−1^) and MNP-PPEGMA10-MTX (C=C at 1641 cm^−1^), respectively, from Figure 1b.

**Figure 2:**
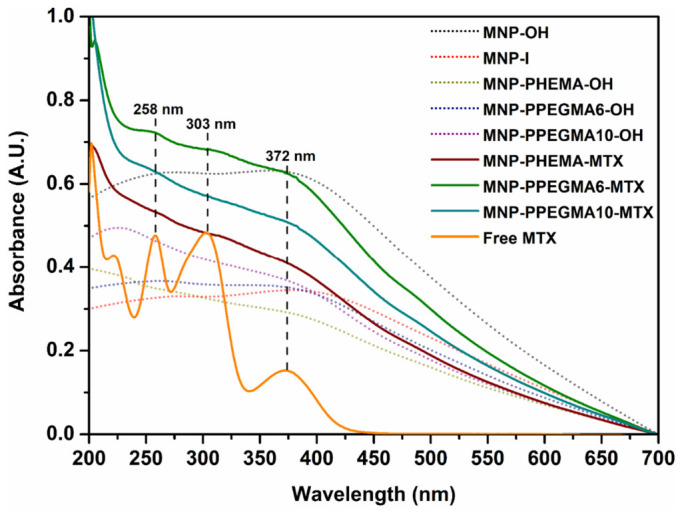
The UV-vis spectra of unmodified magnetic nanoparticles (MNP-OH), decorated with ATRP-type initiator (MNP-I), coated with SI-ATRP-derived polymers (MNP-PHEMA-OH, MNP-PPEGMA6-OH, MNP-PPEGMA10-OH), the corresponding MTX-conjugated nanostructures (MNP-PHEMA-MTX, MNP-PPEGMA6-MTX and MNP-PPEGMA10-MTX), and free MTX.

UV-vis spectroscopy was used to quantify the concentration of MTX covalently bound to the hydroxyl groups on each repetitive structural unit of the polymers. Analyzing Figure 2, unmodified magnetic nanoparticles (MNP-OH) exhibit an absorption maximum at 400 nm, followed by an exponential decrease in the visible range [47,48].

MNP-I exhibited a similar absorption profile, albeit with a slight decrease in the absorbance, due to the ATRP initiator existing on the surface of the nanoparticles and influencing the total absorption of light by the unmodified MNPs. Knowing that poly(2-hydroxyethyl) methacrylate (PHEMA) [49], PPEGMA6 and PPEGMA10 do not show absorption bands in the UV-Vis range, in Figure 2, the samples MNP-PHEMA-OH, MNP-PPEGMA6-OH and MNP-PPEGMA10-OH show the same behavior for the absorption curves as in the case of unmodified MNPs. Free MTX exhibited three main absorption bands in PBS pH 7.4 at 372 nm, 303 nm and 258 nm, with the absorption maximum at 303 nm. The absorption curves of MTX-functionalized MNPs (MNP-PHEMA-MTX, MNP-PPEGMA6-MTX, and MNP-PPEGMA10-MTX) displayed characteristic MTX absorption bands (372 nm, 303 nm and 258 nm) [50]. These signals confirm the successful attachment of MTX onto the nanoparticles coated with the respective polymers. The concentration of MTX in the nanoparticles was determined using a calibration curve of MTX in PBS at pH 7.4 (Figure S2a,b). The determined concentrations of MTX in the nanoparticles were 61.4 µg MTX/mg MNP-PHEMA-MTX, 131 µg MTX/mg MNP-PPEGMA6-MTX, and 58.4 µg MTX/mg MNP-PPEGMA10-MTX.

These values represent the amount of MTX per milligram of the corresponding functionalized MNPs. The determined concentrations reflect the loading capacity of MTX within the polymer-coated nanoparticles and provide quantitative insight into the efficiency of drug incorporation (Table 1).

As shown in Table 1, the drug loading (DL) and encapsulation efficiency (EE) are strongly influenced by the polymer brush architecture. Among the investigated systems, MNP-PPEGMA6-MTX exhibits the highest DL (13.1%) and EE (6.55%), indicating a more favorable balance between polymer chain length and accessibility of functional groups for MTX conjugation. In contrast, both shorter (PHEMA) and longer (PPEGMA10) polymer chains result in lower loading efficiencies, which can be attributed to either limited availability of reactive sites or increased steric hindrance within the polymer corona. These results highlight the critical role of polymer structure in modulating drug loading performance and further support the structure–property relationship governing the behavior of these nanocarriers.

A slight pH-dependent release behavior of MTX was observed (Figure S3), with discernible differences between physiological (pH 7.4) and mildly acidic conditions (pH 5.8). Notably, MNP–PHEMA–MTX and MNP–PPEGMA10–MTX showed a slightly higher release at pH 5.8 compared to pH 7.4, suggesting a partial weakening of drug–matrix interactions under mildly acidic conditions. In contrast, MNP–PPHEMA6–MTX exhibited lower release at pH 5.8, likely due to stronger hydrophobic interactions and reduced methotrexate solubility, limiting diffusion from the matrix. The release profiles at pH 5.8 also displayed a more fluctuating behavior, which can be attributed to the decreased solubility of methotrexate at acidic pH, potentially leading to transient aggregation or re-association phenomena that affect its apparent concentration during quantification. This interpretation is further supported by the incomplete release observed for free methotrexate at pH 5.8, where cumulative values did not reach 100%, most likely due to solubility limitations. Methotrexate is covalently linked to the polymer matrix via an ester bond, which is generally stable in the pH range 5–8 and undergoes significant hydrolysis only under strongly acidic or basic conditions. However, at pH 5.8, a slow and partial ester hydrolysis cannot be excluded, which may contribute to the slightly enhanced drug release observed under mildly acidic conditions. Nevertheless, this contribution is expected to be limited, with the overall release behavior being predominantly governed by polymer–drug interactions and drug solubility. Although a slight pH-dependent release behavior was observed, the overall release at pH 5.8 remains relatively limited, indicating that ester bond cleavage is not significantly accelerated under these conditions. Therefore, the enhanced cytotoxic effects observed in MCF-7 cells cannot be attributed exclusively to a pH-triggered release mechanism. Instead, alternative contributions, such as surface-mediated interactions between the nanocarriers and the cellular membrane, as well as gradual drug availability from the nanoparticle interface, may play a role in the observed biological response. This highlights the complexity of the system and suggests that multiple factors contribute to the overall therapeutic effect.

**Figure 3:**
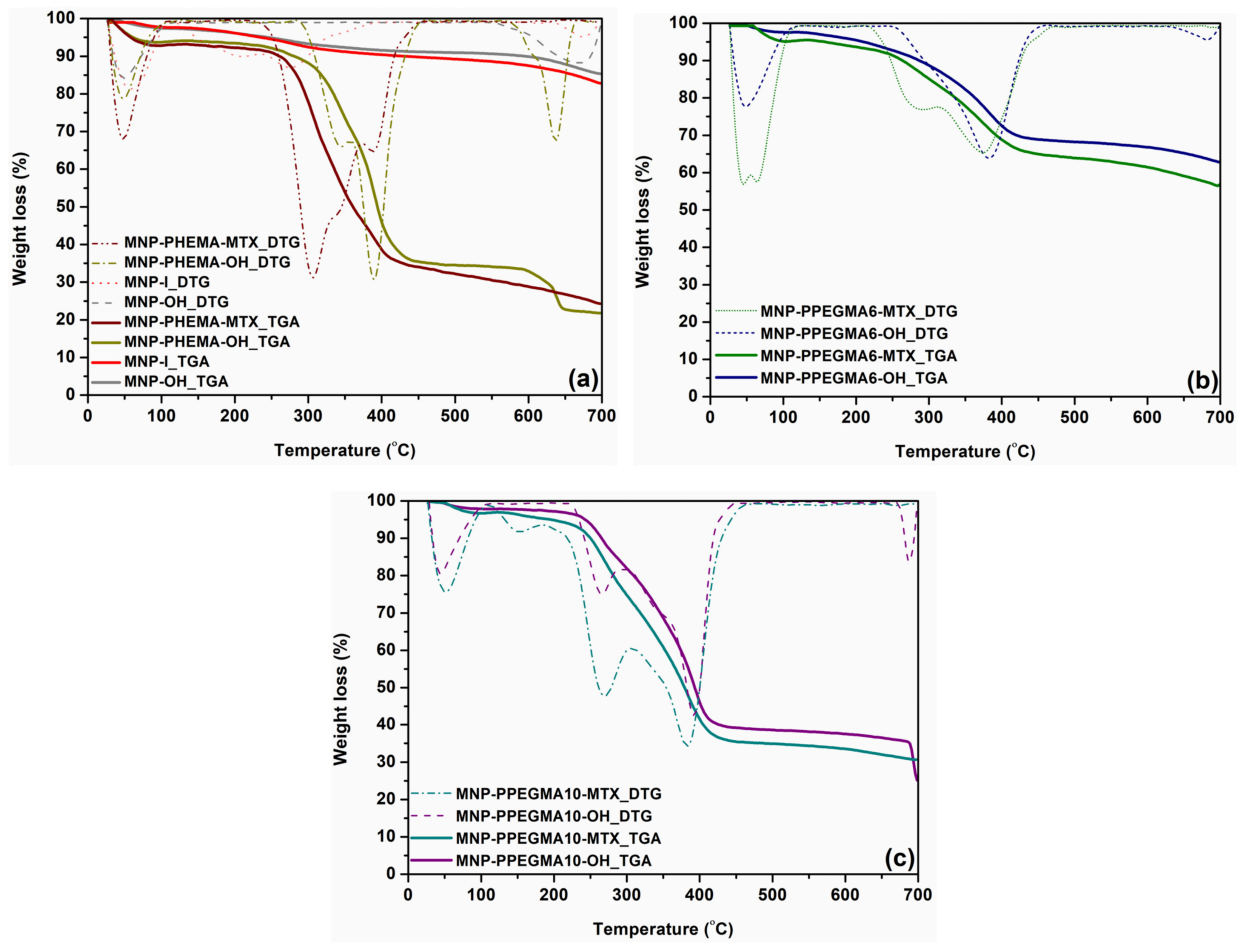
Thermal gravimetric analysis curve (TGA) and the first derivative curve of TGA (DTG) of (**a**) MNP-OH, MNP-I, MNP-PHEMA-OH, MNP-PHEMA-MTX; (**b**) MNP-PPEGMA6-OH, MNP-PPEGMA6-MTX; and (**c**) MNP-PPEGMA10-OH, MNP-PPEGMA10-MTX.

Thermogravimetric analysis (TGA) and derivative curves (DTG) (Figure 3) were used to evaluate the thermal behavior and organic content of the synthesized nanostructures.

For clarity, the key TGA parameters, including weight loss percentages, degradation temperature ranges of organic part loss, and residual mass values, are summarized in Table 2.

All samples showed multi-step degradation behavior. An initial weight loss below 100 °C (Figure 3) is attributed to the removal of physically adsorbed water, followed by a major degradation step associated with the degradation of organic components (initiator, polymer shell, and/or MTX). At higher temperatures, a final stage associated with the decomposition of residual organic fragments is observed. For all samples, the third stage of degradation corresponds to the volatilization of decomposed organic material [51].

As shown in Table 2, polymer-functionalized samples display a substantial increase in organic mass loss compared to unmodified MNPs, confirming the successful growth of polymer brushes via SI-ATRP. This effect is particularly pronounced for PHEMA and PPEGMA10 systems, which exhibit higher overall organic content due to increased polymer contribution.

Following MTX conjugation, a slight increase in the organic fraction is observed for all systems (Table 2). However, these differences remain relatively small, particularly in the case of PPEGMA-based nanostructures. This behavior can be attributed to the moderate MTX loading relative to the total nanoparticle mass, as well as to the overlap between polymer and MTX thermal degradation processes. Additionally, minor shifts in degradation temperature ranges and residual mass values (Table 2) further support changes in the composition of the nanostructures after functionalization.

**Figure 4:**
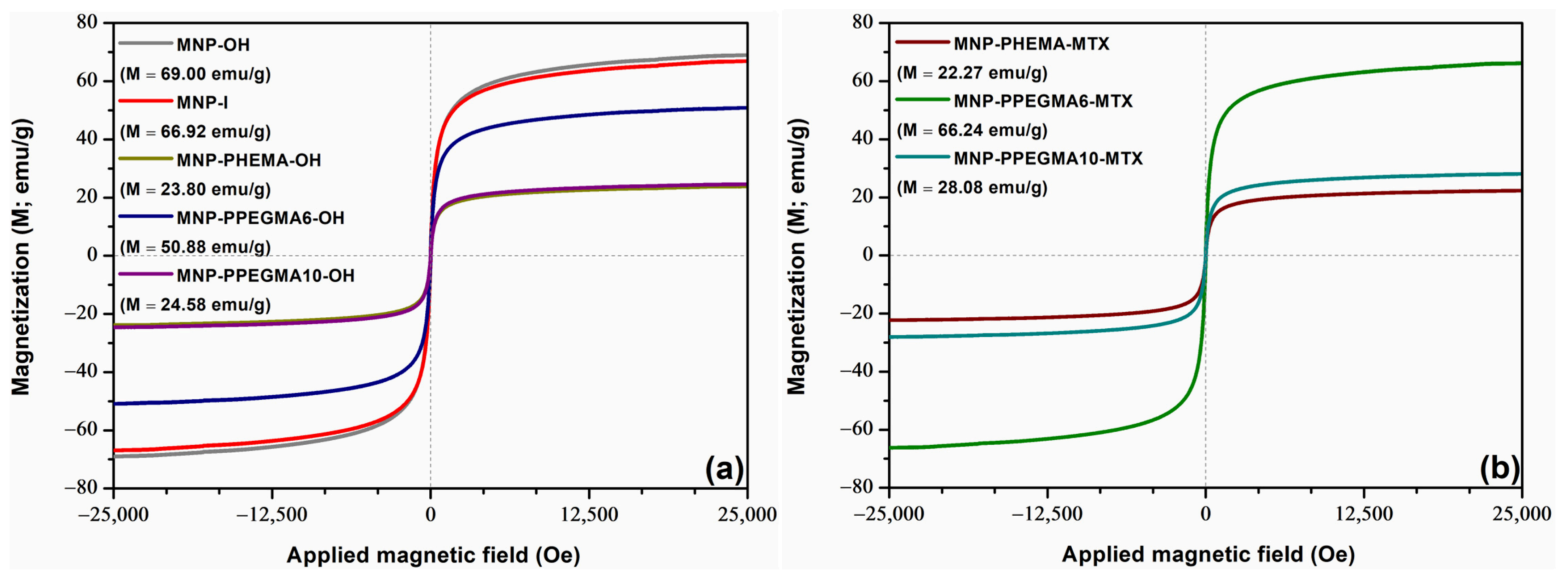
(**a**) VSM magnetization curves of the MNP-OH, MNP-I and polymer-coated samples (MNP-PHEMA-OH, MNP-PPEGMA6-OH and MNP-PPEGMA10-OH) and (**b**) VSM magnetization curves of the MNPs corresponding to MTX-conjugated nanostructures (MNP-PHEMA-MTX, MNP-PPEGMA6-MTX and MNP-PPEGMA10-MTX).

Magnetic characterization confirmed that all formulations retained superparamagnetic behavior (Figure 4a,b). Room-temperature hysteresis loops recorded up to 2.5 T displayed zero coercivity and remanence across the series.

The unmodified magnetic nanoparticles (MNP-OH) showed a saturation magnetization value of 69.00 emu/g. Decorating the surface of these MNPs with the ATRP-type initiator (MNP-I) leads to a slight decrease in the saturation magnetization to the value of 66.92 emu/g. The growth of the three polymers by SI-ATRP on the surface of these MNPs leads to decreases in the saturation magnetization; thus, for the sample MNP-PHEMA-OH, a saturation magnetization value of 23.80 emu/g was obtained, a large decrease compared to the sample MNP-I, which indicates that the polymer grafting density is high [41]. In the case of the MNP-PPEGMA6-OH sample, the saturation magnetization indicated a value of 50.88 emu/g, and the MNP-PPEGMA10-OH sample a value of 24.58 emu/g.

The functionalization of the three polymers grown on the surface of MNPs with MTX leads to changes in the saturation magnetization. Thus, in the case of the MNP-PHEMA-MTX sample, a slight decrease in the saturation magnetization value of 22.27 emu/g is observed. In the case of the MNP-PPEGMA6-MTX and MNP-PPEGMA10-MTX samples, these lead to an increase in magnetization, with 66.24 emu/g in the case of the MNP-PPEGMA6-MTX sample and 28.08 emu/g for the MNP-PPEGMA10-MTX sample. The saturation magnetization values of all the samples in Figure 4a,b fall within the lower limit allowed for magnetic systems that can be used as MRI contrast with a magnetization value of 7 emu/g [52]. The magnetic responsiveness of similar SI-ATRP functionalized SPION systems has been previously demonstrated by our group [41].

**Figure 5:**
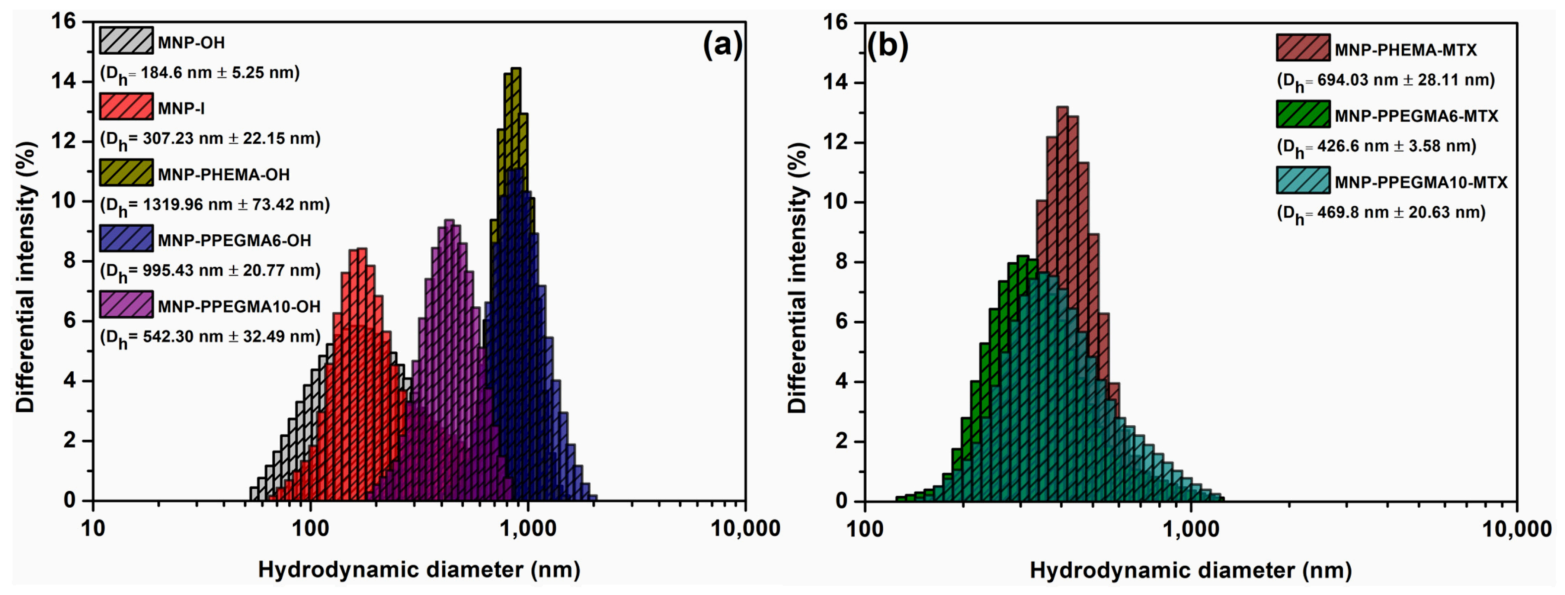
(**a**) The average hydrodynamic diameters of unmodified magnetic nanoparticles (MNP-OH), ATRP-initiator-functionalized nanoparticles (MNP-I) and SI-ATRP-derived polymer-coated nanoparticles (MNP -PHEMA-OH, MNP-PPEGMA6-OH and MNP-PPEGMA10-OH) and (**b**) MTX-conjugated polymer-coated magnetic nanoparticles (MNP-PHEMA-MTX, MNP-PPEGMA6-MTX and MNP-PPEGMA10-MTX).

Dynamic light-scattering (DLS) measurements revealed a step-wise modulation of hydrodynamic size across the synthetic pathway described in Scheme 1 (Figure 5, Table S1).

Unmodified iron oxide nanoparticles (MNP-OH, Figure 5a) exhibited an average hydrodynamic diameter of 184.6 nm ± 5.25 nm with a polydispersity index (PDI) of 0.193 ± 0.005, consistent with literature reports for MNPs synthesized by co-precipitation [41]. Covalent attachment of the ATRP-type initiator onto MNP-OH (MNP-I, Figure 5a) resulted in an increase in hydrodynamic diameter to a value of 307.23 nm ± 22.15 nm, indicating a PDI of 0.200 ± 0.040. For the three SI-ATRP-derived polymers, the average hydrodynamic diameters increase significantly compared to MNP-I, namely, in Figure 5a, for the sample MNP-PHEMA-OH, an average hydrodynamic diameter of 1319.96 nm ± 73.42 nm was determined, with a PDI of 0.422 ± 0.031. MNP-PPEGMA6-OH exhibited an average hydrodynamic diameter of 995.43 nm ± 20.77 nm and a PDI of 0.363 ± 0.008, and the average hydrodynamic diameter of the MNP-PPEGMA10-OH sample was 542.3 nm ± 32.49 nm, with a PDI of 0.227 ± 0.013. After the covalent binding of MTX to the three polymers, the average hydrodynamic diameters of MTX-conjugated polymer-coated nanoparticles decreased compared with the corresponding polymer-coated systems (Figure 5b). Thus, the MNP-PHEMA-MTX sample had an average hydrodynamic diameter of 694.03 nm ± 28.11 nm with a PDI of 0.274 ± 0.012. MNP-PPEGMA6-MTX exhibited an average hydrodynamic diameter of 426.6 nm ± 3.58 nm with a PDI of 0.186 ± 0.0009, and the MNP-PPEGMA10-MTX sample showed an average hydrodynamic diameter of 469.8 nm ± 20.63 nm and a PDI of 0.205 ± 0.003.

**Figure 6:**
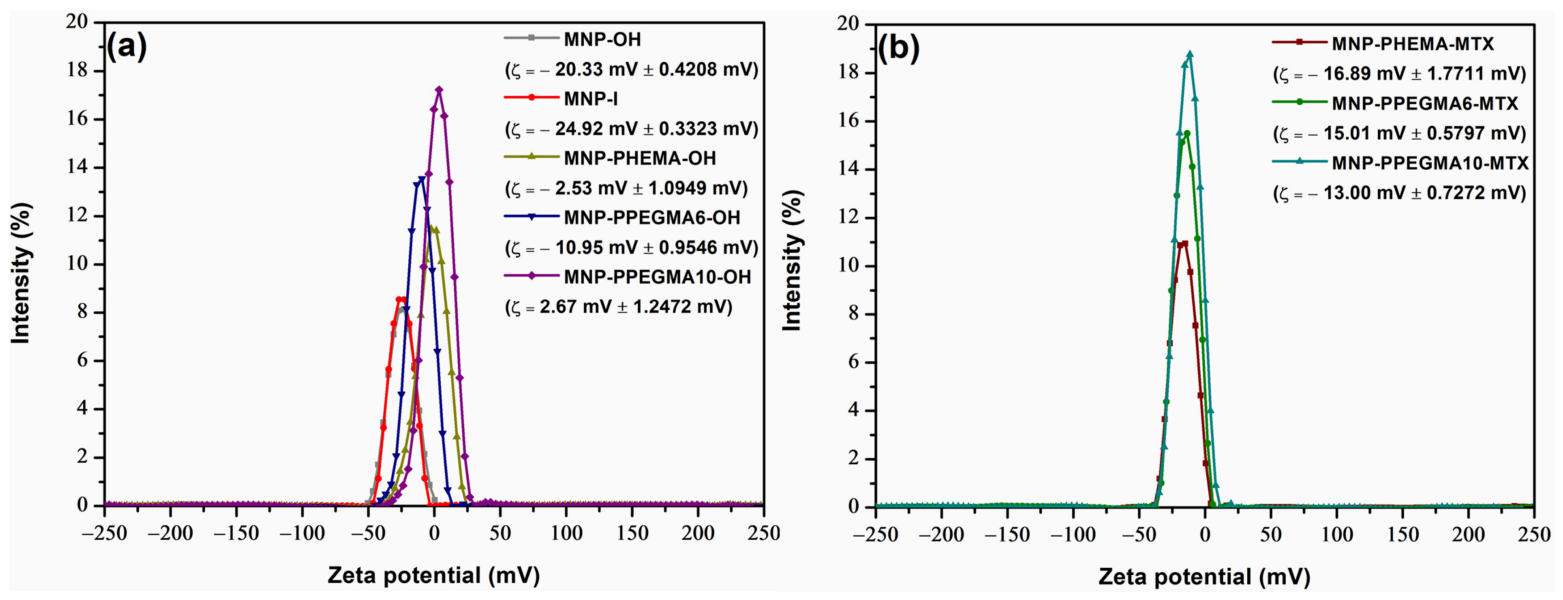
(**a**) Average zeta potentials of unmodified iron oxide nanoparticles (MNP-OH), ATRP initiator (MNP-I) and SI-ATRP-derived polymer-coated nanoparticles (MNP-PHEMA-OH, MNP-PPEGMA6-OH and MNP-PPEGMA10-OH); (**b**) MTX-conjugated polymer-coated magnetic nanoparticles (MNP-PHEMA-MTX, MNP-PPEGMA6-MTX and MNP-PPEGMA10-MTX).

Complementary ζ-potential data (Figure 6, Table S1) reflect these surface transformations and confirm that each formulation maintains sufficient electrostatic repulsion to remain colloidally stable under physiological conditions.

Thus, the unmodified magnetic nanoparticles (MNP-OH, Figure 6a) presented a zeta potential (ζ) of −20.33 mV ± 0.4208 mV, which confirms the fact that, on the surface of these nanoparticles, there are hydroxyl-type groups [53] that indicate a negative potential, and the given value indicates a high colloidal stability [54]. The covalent binding of the ATRP-type initiator (MNP-I, Figure 6a) increases the colloidal stability of the nanoparticles with a value of ζ = −24.92 mV ± 0.3323 mV. For the three SI-ATRP-derived polymers, the zeta potentials of the nanoparticles decrease significantly compared to MNP-I (Figure 6a), resulting in reduced colloidal stability [54], with a ζ = −2.53 mV ± 1.0949 mV for the sample MNP-PHEMA-OH, ζ = 10.95 mV ± 0.9546 mV for MNP-PPEGMA6-OH and ζ = 2.67 mV ± 1.2472 mV for the MNP-PPEGMA10-OH sample. The reduced colloidal stability observed for these samples was improved after covalent MTX conjugation by the free hydroxyl groups on the side chains of the polymeric repeating units (Figure 6b), resulting in moderate colloidal stability of the nanocarriers [54] (ζ = −16.86 mV ± 1.7711 mV for the MNP-PHEMA-MTX sample, ζ = −15.01 mV ± 0.5797 mV in the case of the MNP-PPEGMA6-MTX sample and ζ = −13.00 mV ± 0.7272 mV for the MNP-PPEGMA10-MTX sample).

**Figure 7:**
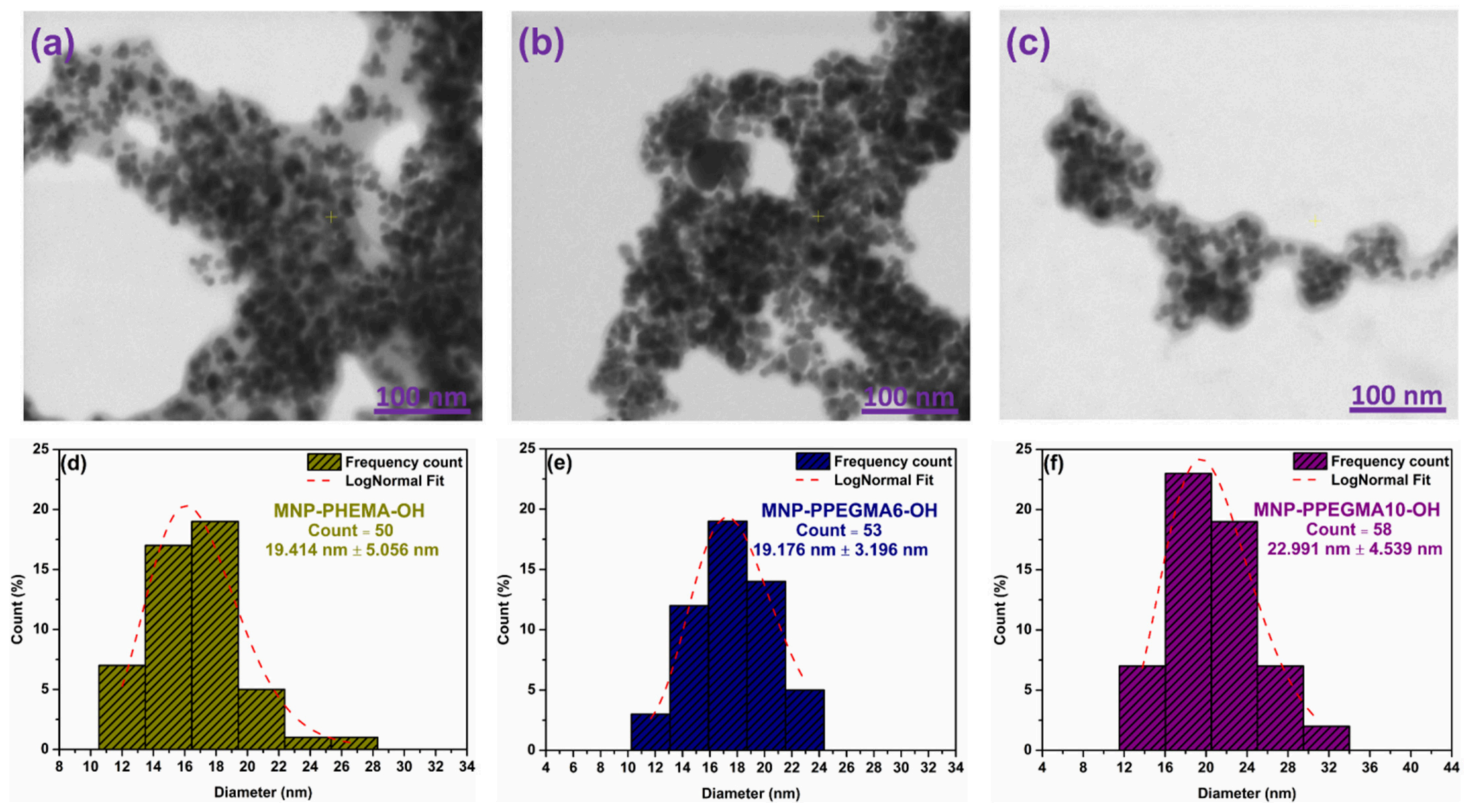
STEM images and particle size distribution histogram of SI-ATRP-derived polymer-coated MNPs (MNP-PHEMA-OH: (**a**,**d**); MNP-PPEGMA6-OH: (**b**,**e**); and MNP-PPEGMA10-OH: (**c**,**f**)).

The scanning transmission electron microscopy (STEM) technique was used to evaluate the morphology and dimensions of the nanoparticles, including unmodified nanoparticles, initiator-coated systems, polymer-coated samples, and MTX-conjugated formulations. Analysis of Figure S4 revealed that, in the case of unmodified magnetic nanoparticles (MNP-OH, Figure S4a,c) and those with ATRP-type initiator grafted on the surface (MNP-I, Figure S4b,d), there was a tendency toward aggregation, mainly due to solvent evaporation after deposition on the TEM grid, exhibiting spherical morphology, with an average diameter of 10.367 nm ± 2.019 nm for the MNP-OH sample and 13.162 nm ± 1.759 nm in the case of the MNP-I sample. Following SI-ATRP polymer growth, the nanoparticles exhibited a core-shell morphology with larger diameters compared to MNP-OH and MNP-I (the core being due to the MNPs and the shell coming from the polymers). These structures were observed as agglomerated, formed after solvent removal. Thus, the MNP-PHEMA-OH sample (Figure 7a,d) exhibited an average diameter of 19.414 nm ± 5.056 nm, with an average diameter of 19.176 nm ± 3.196 nm in the case of the MNP-PPEGMA6-OH sample (Figure 7b,e), and an average diameter of 22.991 nm ± 4.539 nm for the MNP-PPEGMA10-OH sample (Figure 7c,f).

**Figure 8:**
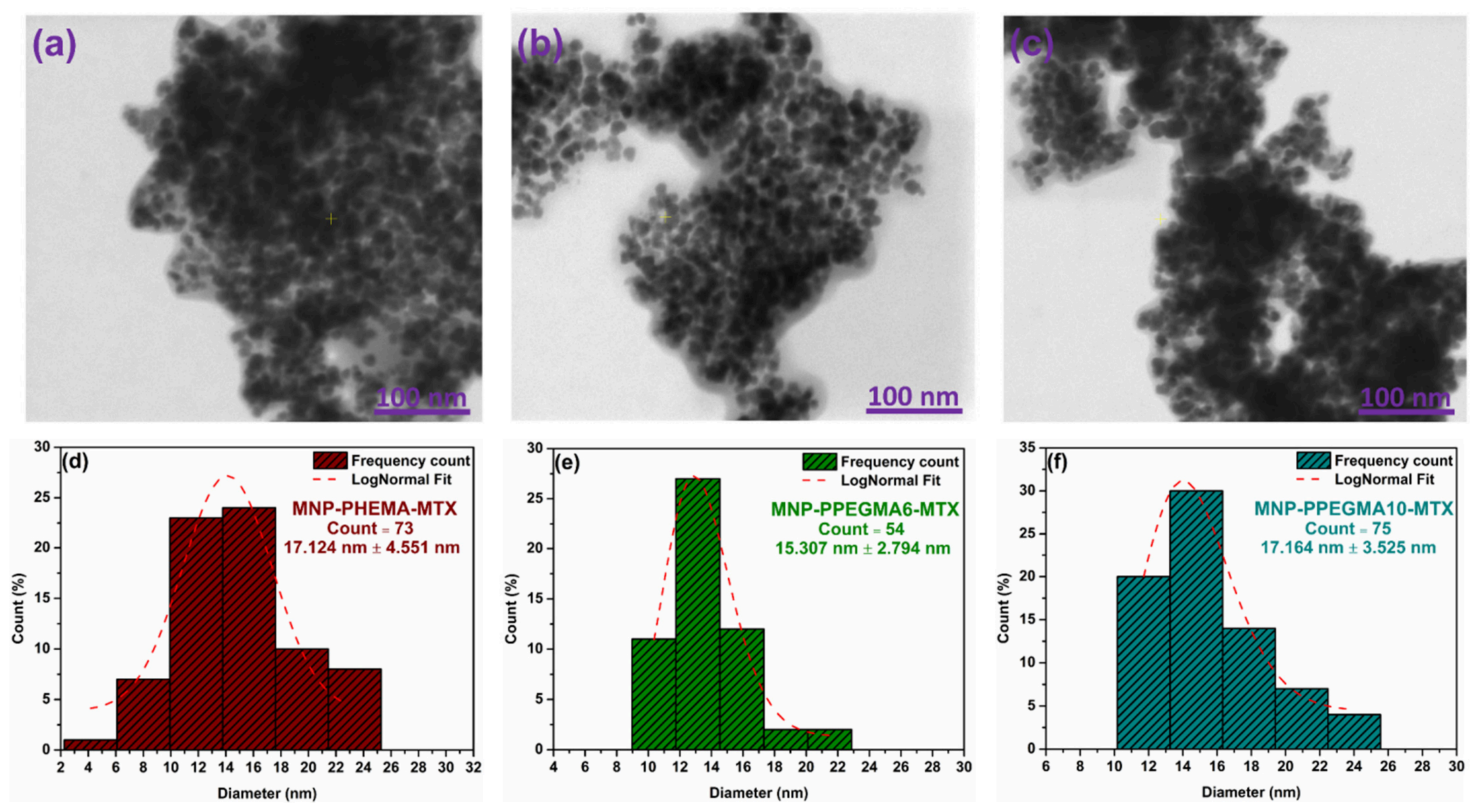
STEM images and particle size distribution histogram of MNPs coated with MTX-functionalized polymers (MNP-PHEMA-MTX: (**a**,**d**); MNP-PPEGMA6-MTX: (**b**,**e**); MNP-PPEGMA10-MTX: (**c**,**f**).

MTX conjugation resulted in reduced average diameters compared to nanoparticles with polymers grown on the surface; this is likely because, by covalently introducing methotrexate on the side chains of the polymers, polymer swelling is reduced, resulting in smaller diameters for MTX-conjugated samples compared with non-functionalized systems [41]. A similar trend was observed for the hydrodynamic diameters determined by DLS. Thus, the MNP-PHEMA-MTX sample (Figure 8a,d) exhibited an average diameter of 17.124 nm ± 4.551 nm, with 15.307 nm ± 2.794 nm for MNP-PPEGMA6-MTX (Figure 8b,e) and 17.164 nm ± 3.525 nm for the MNP-PPEGMA10-MTX sample (Figure 8c,f).

**Figure 9:**
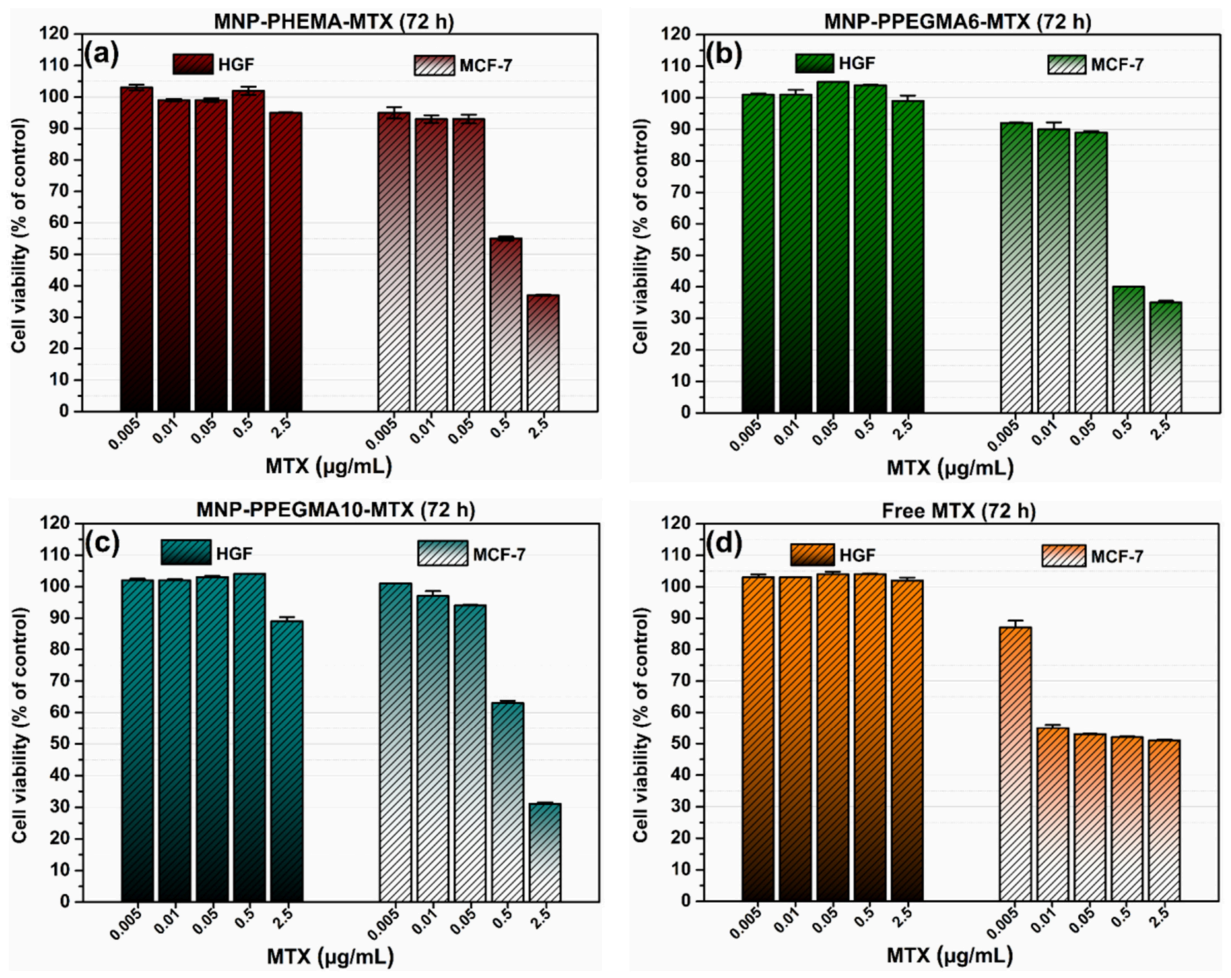
Cytotoxicity of MNPs coated with MTX-functionalized polymers: MNP-PHEMA-MTX (**a**), MNP-PPEGMA6-MTX (**b**), MNP-PPEGMA10-MTX (**c**), and free MTX (**d**).

Cell viability was assessed using the CellTiter-Glo^®^ assay after 72 h incubation with human gingival fibroblasts (HGF, Figure S5) and MCF-7 breast adenocarcinoma cells (Figure 9). Polymer-coated MNPs lacking MTX (MNP-PHEMA-OH, MNP-PPEGMA6-OH, and MNP-PPEGMA10-OH) proved fully biocompatible, maintaining >95% HGF viability across all tested concentrations. Covalent attachment of MTX preserved this benign profile in normal cells while triggering a pronounced, dose-dependent cytotoxic effect in MCF-7 cells, reducing viability by up to 69% at 2.5 µg MTX/mL. Free MTX, in contrast, induced a pronounced cytotoxic effect, particularly at lower concentrations, indicating rapid drug action. However, the MTX-loaded nanostructures exhibited a more gradual and controlled cytotoxic response, which can be attributed to the covalent attachment of MTX and its gradual availability.

These results highlight that the advantage of nanoparticle-mediated delivery does not rely solely on maximizing immediate cytotoxicity, but rather on providing controlled drug exposure and improved selectivity. Importantly, all MTX-functionalized systems showed minimal toxicity toward fibroblasts, suggesting a favorable safety profile. The observed cytotoxic effect is consistent with the role of MTX when incorporated into functionalized polymer-coated MNPs, being internalized through folate receptors overexpressed on tumor cells compared to healthy ones [27], leading to inhibition of cell proliferation. This behavior differs from our previous results using folic acid as a targeting moiety, which promoted nanoparticle internalization but did not induce MCF-7 cell death [41].

Furthermore, MTX-functionalized nanoparticles induce a clear dose-dependent cytotoxic response in MCF-7 cells at concentrations higher than 0.05 µg/mL. Among the three MTX formulations, MNP-PPEGMA6-MTX exhibited the greatest tumor-selective potency. This outcome aligns with its physicochemical attributes: the intermediate-length PPEGMA6 brush supports the highest drug loading (131 µg/mg), achieves a compact hydrodynamic diameter of 427 nm post-conjugation, and retains a ζ-potential (~−30 mV) that promotes colloidal stability and cellular uptake. The shorter PHEMA and longer PPEGMA10 brushes, which bear lower MTX densities and/or larger hydrated diameters, produced more modest cytotoxic responses. In addition, the ability to tailor the polymer brush architecture and drug loading highlights the versatility of this nanoplatform, which can be extended to other therapeutic agents, supporting the rational design of multifunctional nanocarriers for targeted drug delivery applications.

### Study Limitations

While the present study demonstrates promising physicochemical properties and in vitro biological activity of the developed nanocarriers, several limitations should be acknowledged. First, the observed selective cytotoxicity toward MCF-7 cells, although encouraging, remains preliminary and indirect. The mechanism of cellular internalization and intracellular trafficking was not investigated in this study and, therefore, remains speculative in the absence of dedicated imaging techniques such as confocal microscopy or transmission electron microscopy (TEM). Second, the colloidal stability of the nanoparticles was evaluated only in PBS under controlled conditions. Stability in complex biological media containing serum proteins (e.g., FBS) was not assessed, which represents an important limitation, particularly for PEGylated systems where protein adsorption and corona formation may significantly affect biological behavior. Finally, although all formulations exhibited superparamagnetic behavior, magnetic guidance, targeting, and localization were not experimentally validated. Therefore, any implications regarding their use in magnetically guided delivery remain theoretical and were not experimentally demonstrated. These aspects highlight the need for further studies to better understand the biological mechanisms and translational applicability of the proposed nanoplatform.

## 4. Conclusions

This study presents a clear structure–property relationship for superparamagnetic iron oxide nanoparticles coated with poly(methacrylate) brushes synthesized via surface-initiated atom-transfer radical polymerization. The dopamine-anchored initiator enabled the formation of dense, conformal grafts without substantially compromising the magnetic saturation required for MRI contrast and magnetic manipulation. Systematic variation of brush length revealed that an intermediate poly(poly(ethylene glycol) methacrylate) architecture (PPEGMA6) maximized methotrexate (MTX) payload (131 µg/mg), promoted favorable compaction of the hydrated shell, and preserved a ζ-potential conducive to colloidal stability and cellular uptake. Correlative physicochemical and biological analyses demonstrated that MTX conjugation produces adaptive reorganization of the polymer shell, reducing the hydrodynamic diameter while maintaining superparamagnetic behavior. Importantly, all MTX-decorated formulations remained benign toward primary human fibroblasts yet produced strong, dose-dependent cytotoxicity in MCF-7 breast cancer cells, with the PPEGMA6 design achieving a 69% reduction in viability, outperforming free MTX at equivalent concentrations. The presented data highlight the significant influence of polymer brush architecture on drug loading efficiency, nanoscale colloidal behavior, and therapeutic index. The PPEGMA6-MTX nanocarrier, therefore, emerges as a promising platform for further biological evaluation and the development of magnetically responsive drug-delivery systems. More broadly, the SI-ATRP methodology described herein provides a versatile platform for the rational design of next-generation nanoparticle bearing diverse pharmacophores and targeting ligands.

## Figures and Tables

**Table 1:** Drug loading (DL) and encapsulation efficiency (EE) of MTX in functionalized MNPs.

Sample Name	DL (%)	EE (%)
MNP-PHEMA-MTX	6.14	3.07
MNP-PPEGMA6-MTX	13.1	6.55
MNP-PPEGMA10-MTX	5.84	2.92

**Table 2:** Summary of key thermogravimetric parameters for the analyzed samples.

Sample	Water Loss (%)	Organic Loss (%)	Temperature Range of Organic Part Loss (°C)	T_max_ (°C)	Residual Mass (%)
MNP-OH	2.37	6.44	135–427	279	85.89
MNP-I	2.24	8.75	154–571	272	83.60
MNP-PHEMA-OH	5.51	57.77	251–594	390	24.27
MNP-PHEMA-MTX	7.43	67.01	261–406	45	25.44
MNP-PPEGMA6-OH	2.35	30.56	139–419	391	62.90
MNP-PPEGMA6-MTX	5.99	31.19	247–420	375	57.04
MNP-PPEGMA10-OH	3.58	60.38	232–419	390	25.96
MNP-PPEGMA10-MTX	3.45	65.11	139–412	383	31.14

## Data Availability

All data generated or analyzed during this study are included in this published article and its Supplementary Materials.

## Supplementary Materials

The following supporting information can be downloaded at: https://www.mdpi.com/article/10.3390/pharmaceutics18060691/s1: Figure S1: FTIR spectra of the 2-hydroxyethyl methacrylate (HEMA), poly(ethylene glycol) methacrylate with average Mn = 360 g/mol (PEGMA6) and poly(ethylene glycol) methacrylate, with an average Mn = 500 g/mol (PEGMA10). A.U.—arbitrary units.; Figure S2: Absorption spectra of MTX solutions of different concentrations (0.2–10 μg/mL) in PBS with pH = 7.4 (a) and 5.8 (c), and calibration curve of MTX in PBS with pH = 7.4 (b) and pH = 5.8 (d) measured at λ = 303 nm. A.U.—arbitrary units.; Figure S3: Time-dependent cumulative release profiles of methotrexate (MTX) from MNP-based systems and free MTX at (a) pH 7.4 and (b) pH 5.8 (37 °C).; Figure S4: STEM images and the histogram of particle size distribution of unmodified magnetic nanoparticles (MNP-OH, a and c) and with the grafted ATRP-type initiator on the surface of magnetic nanoparticles (MNP-I, b and d).; Figure S5: Biocompatibility of MNP-PHEMA-OH (a), MNP-PPEGMA6-OH (b) and MNP-PPEGMA10-OH (c) on human gingival fibroblasts (HGF) after 72 h. The experiments were carried out in triplicate, and the viability of treated cells was expressed as a percentage of the viability of control cells. Data are represented as means ± standard error of the mean.; Table S1: The hydrodynamic diameter (D_h_) and the zeta potential (ζ) measured by DLS of MNP-OH, MNP-I, MNP-PHEMA-OH, MNP-PPEGMA6-OH, MNP-PPEGMA10-OH, MNP-PHEMA-MTX, MNP-PPEGMA6-MTX and MNP-PPEGMA10-MTX in 1X PBS pH = 7.4 at a concentration of 50 μg/mL.
