# Supplementary material for: Versatile SI-ATRP Growth of Methacrylate Brushes on Superparamagnetic Iron Oxide Nanoparticles Enables Methotrexate-Mediated Antineoplastic Activity in MCF-7 Cells
Source: Pharmaceutics. 2026 Jun 1;18(6):691. doi: 10.3390/pharmaceutics18060691 (PMC13306494; doi:10.3390/pharmaceutics18060691)
Supplement: Supplementary file 1 [file pharmaceutics-18-00691-s001.zip › pharmaceutics-4219249-supplementary.pdf]

Supplementary Materials: Versatile SI-ATRP Growth of Methacrylate Brushes on Superparamagnetic Iron Oxide Nanoparticles Enables Methotrexate-Mediated Antineoplastic Activity in MCF-7 Cells

Razvan Ghiarasim, Alexandru Rotaru, Cristian-Dragos Varganici, Mariana Pinteala, Narcisa-Laura Marangoci, Ion Tiginyanu and Natalia Simionescu

S1. Fourier-transform infrared spectroscopy spectra of three methacrylate-type monomers

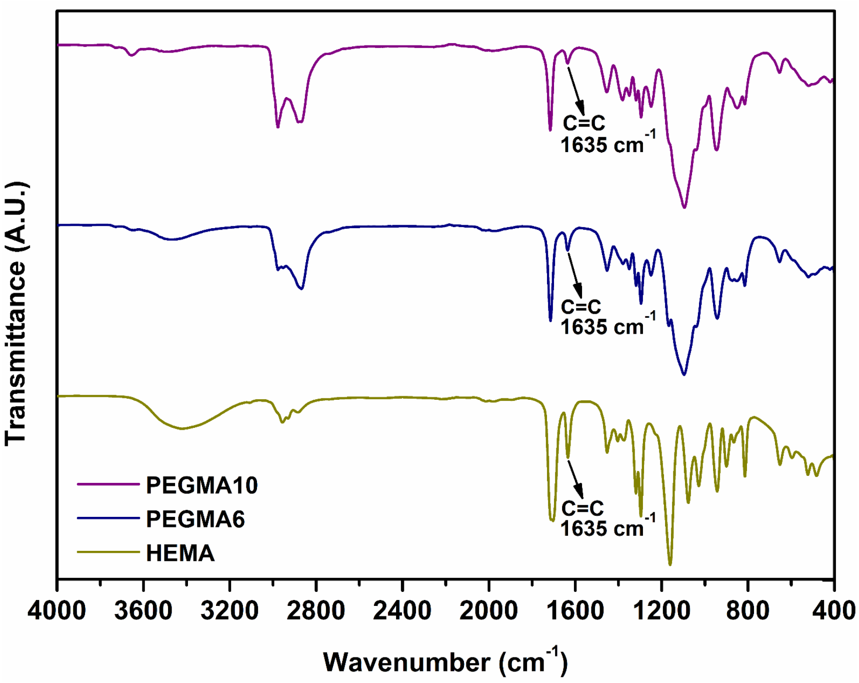

Figure S1. FTIR spectra of the 2-hydroxyethyl methacrylate (HEMA), poly(ethylene glycol) methacrylate with average M_n = 360 g/mol (PEGMA6) and poly(ethylene glycol) methacrylate with average M_n = 500 g/mol (PEGMA10). A.U.-arbitrary units.

S2. UV-Vis calibration curves and in vitro release profiles of methotrexate in PBS at pH 7.4 and 5.8, illustrating pH-dependent behaviour

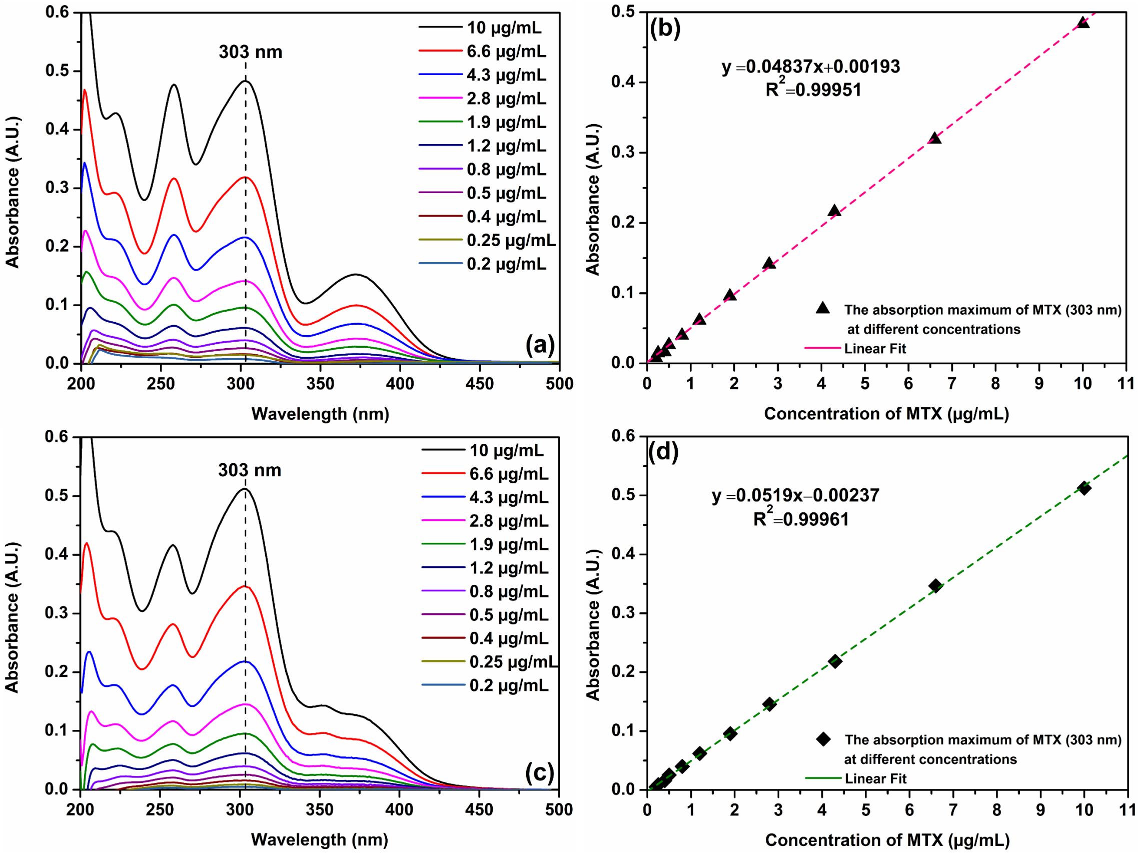

Figure S2. Absorption spectra of MTX solutions of different concentrations (0.2 - 10 μg / mL) in PBS with pH = 7.4 (a), 5.8 (c) and calibration curve of MTX in PBS with pH = 7.4 (b) and pH = 5.8 (d) measured at λ = 303 nm. A.U.-arbitrary units.

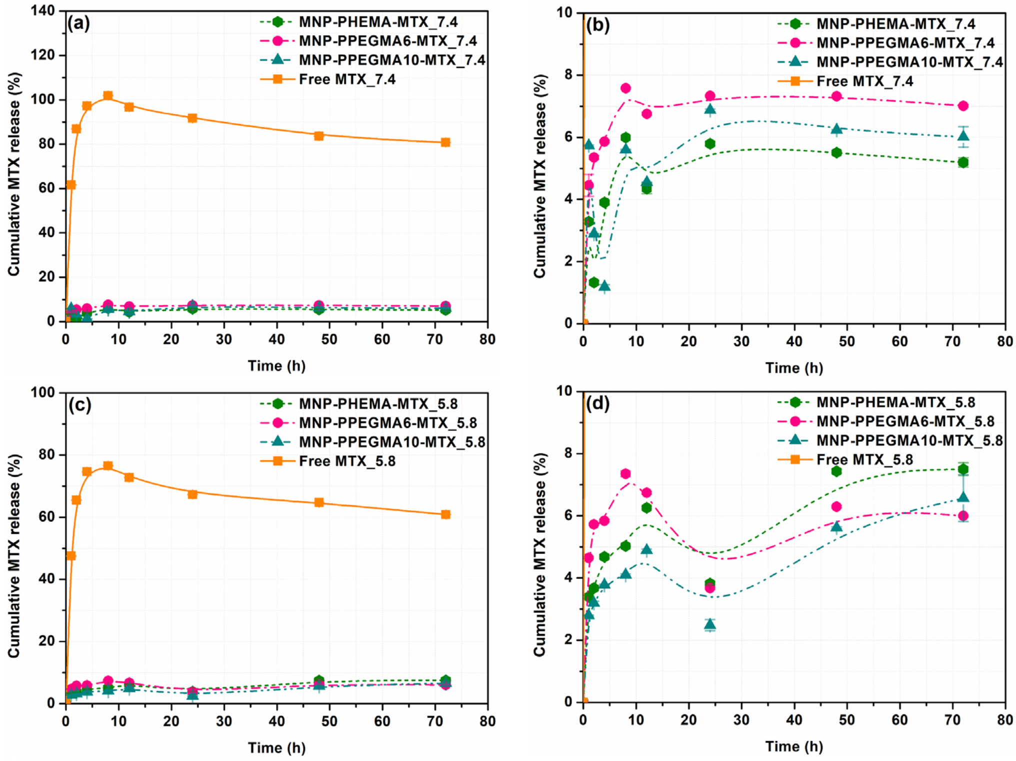

Figure S3. Time-dependent cumulative release profiles of methotrexate (MTX) from MNP-based systems and free MTX at (a) pH 7.4 and (b) pH 5.8 (37 °C).

S3. Hydrodynamic Diameter (D_h) and Zeta Potential (ζ)

Table S1. The hydrodynamic diameter (D_h) and the zeta potential (ζ) measured by DLS of MNP-OH, MNP-I, MNP-PHEMA-OH, MNP-PPEGMA6-OH, MNP-PPEGMA10-OH, MNP-PHEMA-MTX, MNP-PPEGMA6-MTX and MNP-PPEGMA10-MTX in PBS pH=7.4 at concentration of 50 μg/mL.

Sample	D_h (nm)	Average D_h (nm ± SD*)	PDI* z (mV)	Average PDI* ± SD	z (mV)	Average z ± SD*
**MNP-OH**	177.4	184.6 ± 5.25	0.183	0.193 ± 0.005	-20.00	-20.33 ± 0.4208
	186.6		0.197		-20.08	
	189.8		0.193		-20.34	
**MNP-I**	299.1	307.23 ± 22.15	0.221	0.200 ± 0.040	-24.45	-24.92 ± 0.3323
	285.1		0.236		-25.16	
	337.5		0.144		-25.15	
**MNP-PHEMA-OH**	1348.2	1319.96 ± 73.42	0.435	0.422 ± 0.031	-2.59	-2.53 ± 1.0949
	1392.4		0.453		-3.84	
	1219.3		0.380		-1.16	
**MNP-PPEGMA6-OH**	966.3	995.43 ± 20.77	0.352	0.363 ± 0.008	-9.90	-10.95 ± 0.9546
	1006.7		0.373		-12.21	
	1013.3		0.365		-10.74	
**MNP-PPEGMA10-OH**	525.1	542.3 ± 32.49	0.219	0.227 ± 0.013	1.34	2.67 ± 1.2472
	514.0		0.217		4.34	
	587.8		0.246		2.34	
**MNP-PHEMA-MTX**	681.4	694.03 ± 28.11	0.271	0.274 ± 0.012	-15.91	-16.89 ± 1.7711
	733.0		0.292		-15.39	
	667.7		0.261		-19.38	
**MNP-PPEGMA6-MTX**	422.8	426.6 ± 3.58	0.188	0.186 ± 0.0009	-14.20	-15.01 ± 0.5797
	425.6		0.186		-15.33	
	431.4		0.186		-15.51	
**MNP-PPEGMA10-MTX**	471.1	469.8 ± 20.63	0.207	0.205 ± 0.003	-13.04	-13.00 ± 0.7272
	443.9		0.200		-12.09	
	494.2		0.208		-13.87	

S4. Scanning transmission electron microscopy (*STEM*)

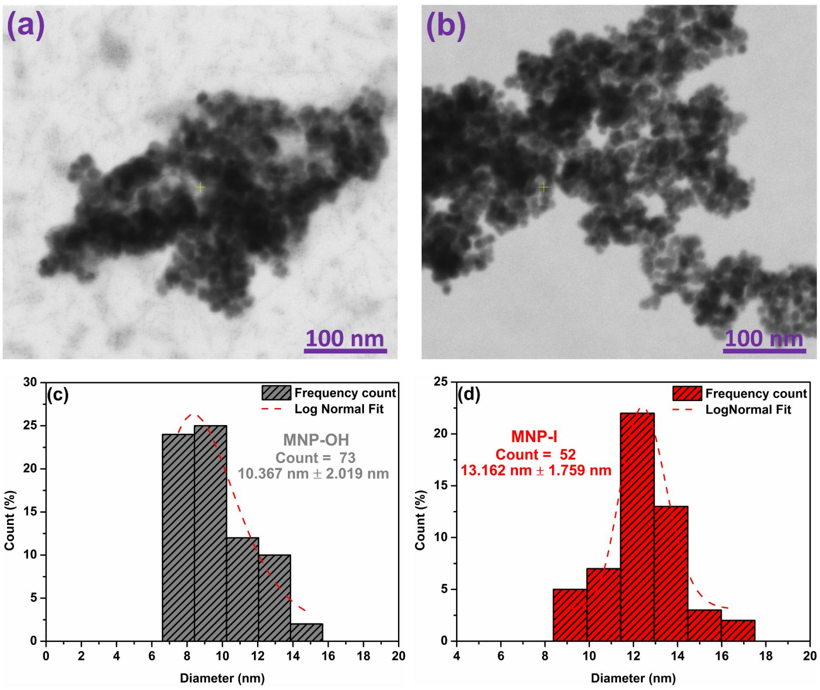

Figure S4. STEM images and the histogram of particle size distribution of unmodified magnetic nanoparticles (MNP-OH, a and c) and with the grafted ATRP-type initiator on the surface of magnetic nanoparticles (MNP-I, b and d).

S5. Biocompatibility of MNP-PHEMA-OH, MNP-PPEGMA6-OH and MNP-PPEGMA10-OH on human gingival fibroblasts (HGF) after 72 h

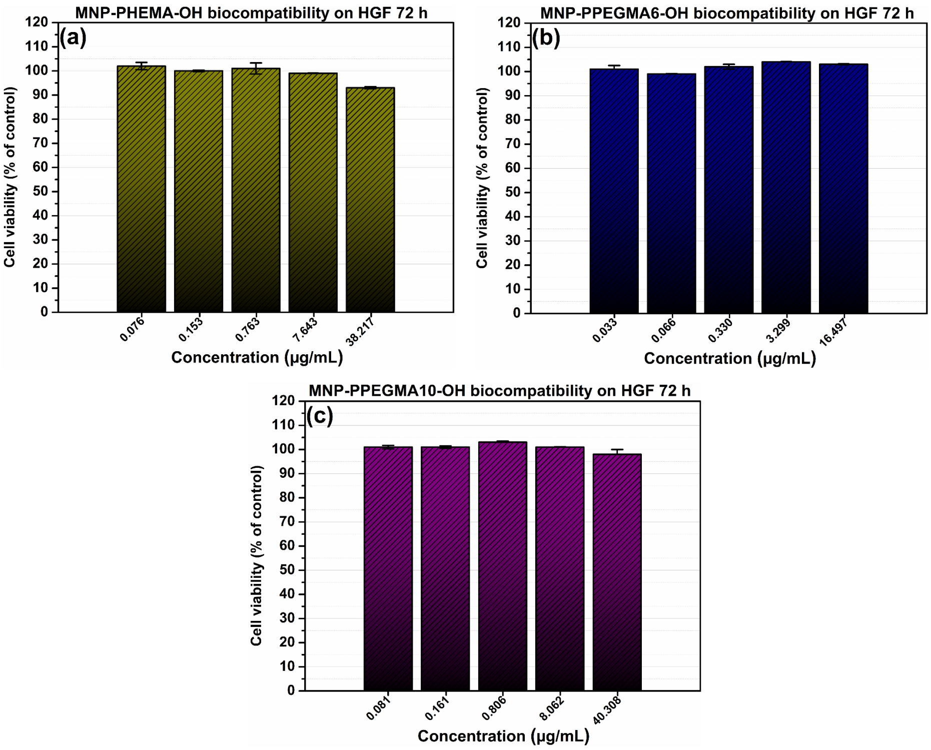

Figure S5. Biocompatibility of MNP-PHEMA-OH (a), MNP-PPEGMA6-OH (b) and MNP-PPEGMA10-OH (c) on human gingival fibroblasts (HGF) after 72 h. The experiments were carried out in triplicate, and the viability of treated cells was expressed as a percentage of the viability of control cells. Data were represented as means ± standard error of the mean.
